# Constant compression decreases vascular bud and VEGFA expression in a rabbit vertebral endplate ex vivo culture model

**DOI:** 10.1371/journal.pone.0234747

**Published:** 2020-06-25

**Authors:** Jia-Wen Zhan, Shang-Quan Wang, Min-Shan Feng, Xu Wei, Jie Yu, Xun-Lu Yin, Tao Han, Li-Guo Zhu

**Affiliations:** 1 General Orthopedics Department, Wangjing Hospital, China Academy of Chinese Medical Sciences, Beijing, China; 2 Key Laboratory of Beijing of Palasy Technology, Wangjing Hospital, China Academy of Chinese Medical Sciences, Beijing, China; 3 Spine Department 2, Wangjing Hospital, China Academy of Chinese Medical Sciences, Beijing, China; 4 Scientific Research Office, Wangjing Hospital, China Academy of Chinese Medical Sciences, Beijing, China; Washington University in Saint Louis, UNITED STATES

## Abstract

**Summary of background data:**

The vascular buds in the vertebral endplate (VEP) are the structural foundation of nutrient exchange in the intervertebral disc (IVD). VEGF is closely related to angiogenesis in the endplate and intervertebral disc degeneration (IDD).

**Objective:**

To investigate the effects of static load on vascular buds and VEGF expression in the VEP and to further clarify the relation between IDD and VEGF.

**Methods:**

IVD motion segments were harvested from rabbit lumbar spines and cultured under no-loading conditions (controls) or in custom-made apparatuses under a constant compressive load (0.5 MPa) for up to 14 days. Tissue integrity and the number of vascular buds were determined, and the concentrations and expression of Aggrecan, COL2a1, and VEGFA in the VEPs were assessed after 3, 7, and 14 days of culturing and then compared with those of fresh tissues.

**Results:**

Under the constant compression, the morphological integrity of the VEPs was gradually disrupted, and immunohistochemistry results showed a significant decrease in the levels of Agg and COL2a1. During the static load, the number of vascular buds in the VEPs was gradually reduced from the early stage of culture, and ELISA showed that the constant compressive load caused a significant decrease in the VEGFA and VEGFR2 protein concentrations, which were consistent with the immunohistochemistry results. Western blot and RT-PCR results also showed that the loading state caused a significant decrease in VEGFA expression compared with that of fresh and control samples.

**Conclusions:**

Constant compression caused degeneration of the VEP as well as a decreased number of vascular buds, thereby accelerating disc degeneration. VEGFA is involved in this process. We anticipate that regulating the expression of VEGFA may improve the condition of the lesions to the vascular buds in the endplates, thus enhancing the nutritional supply function in IVD and providing new therapeutic targets and strategies for the effective prevention and treatment of IDD.

## Introduction

Intervertebral disc degeneration (IDD) is an essential pathological process that causes degenerative spinal disease. The objectives of new biological therapies to treat this type of disease are to prevent or delay IDD and to promote repair, relieve symptoms, or at minimum, delay the need for surgery[1]. For these reasons, determining the mechanism underlying the biological and mechanical pathways that lead to IDD is essential for developing biological therapies and the study of degenerative spinal disease.

The vertebral endplate (VEP) is a structure that is located at the junction between the upper or lower vertebral bodies (VBs) and the adjacent intervertebral disc (IVD). The primary functions of the VEP are mechanical conduction and nutrient transportation to the IVD[2, 3]. In recent years, the role of the endplate in IDD, which is closely related to the nutritional supply to the IVD, has received extensive attention[4, 5]. Nguyen[6] showed that the VEP includes the cartilage endplate (CEP) and bony endplate (BEP), and ultrastructural research found dense capillary loops, known as vascular buds, in the BEP underneath the CEP. The number and function of these vascular buds determine the permeability of the vertebral bone-vertebral endplate-IVD interfaces[7, 8]. Moreover, studies have reported a difference in the vascular architecture between the superior and inferior VEPs[9], and that there are more vascular buds distributed in the cranial endplate than in the caudal endplate[10]. At present, it is believed that the nutritional supply to the IVD comes primarily from diffusion through the vascular buds and the endplate path in the bone marrow contact channel[5, 11]. Therefore, the vascular buds in the VEP are the structural foundation for nutrient exchange in the IVD. Studies have shown that the initiating factors of IDD are disturbances in nutrition metabolism and transport functions, which can be induced by a reduced supply of vascular buds caused by an endplate lesion under abnormal loading[12, 13]. Therefore, investigating the mechanisms underlying IVD injury and regulation is of great importance to preventing or slowing the progression of IDD.

A variety of cytokines regulate the physiological functions of VEP, and VEGF is closely related to angiogenesis in the VEP[14]; however, the role of VEGF in IDD remains controversial. Some studies have suggested that VEGF can increase the number of vascular buds in the VEP and delay the calcification[15]. However, other studies have shown that VEGF can aggravate the inflammation that marks the beginning of IDD[16]. Thus, determining the molecular mechanism underlying the interaction between VEGF and IDD is of significance for the treatment of related diseases. In this study, an *ex vivo* cultured rabbit motion segment was used to observe the effects of constant compressive load on vascular buds and VEGF expression in the vertebral endplate, and to establish the relationship between IDD and VEGF.

Unlike the *in vivo* models, which lack both close control and easy monitoring of complex metabolisms and signalling cascades, *ex vivo* culturing systems are very appealing. These systems allow for better control of the underlying biochemical and biomechanical factors[17]. *Ex vivo* organ models offer an experimental basis to observe the responses and changes in IVD tissues to specific external stimuli[18–22], such as mechanical loads[23, 24] or biologics[25, 26]. Studies have shown that compared with large animal IVDs, the surface ratio of discs in small animals is suitable for the diffusion of nutrients and metabolites[27–29]; thus, this model is more suitable for the observation of long-term intervention on degenerative discs *ex vivo*. Relevant study indicators showed that IVDs from the New Zealand rabbit were more stable in *ex vivo* culture for biomechanics research than other IVDs. Accordingly, we speculate that IDD is related to vascular bud injury in the VEP caused by an abnormal load, which results in the dysfunction of nutrition and metabolism of IVD, and VEGF within the VEP is involved in this process. We use the rabbit motion segment as an ex vivo model in the current mechanobiological study.

## Materials and methods

### 1.1 Animals and tissue samples

All experimental procedures were conducted following the institutional guidelines for the care and use of laboratory animals of the China Academy of Chinese Medical Sciences, Beijing, China. The experimental animals included 112 eighteen-week-old (3.0 kg) male New Zealand White rabbits. These animals were used in accordance with protocols approved by the animal ethics committee of the Institute of Basic Theory for Chinese Medicine, China Academy of Chinese Medical Sciences (Approval No. 20180406). The animals were anaesthetized with pentobarbital (100 mg/kg). Five minutes before death, 25,000 IU heparin was administered through the ear vein, and the lumbar spine was harvested under sterile conditions immediately after euthanasia via CO_2_ asphyxiation.

### 1.2 Method

#### 1.2.1 Sampling and culture

Based on the initial sampling method[30], soft tissues and posterior elements of the spine were removed, and only one motion segment (L4/5) per rabbit was used for testing. These segments consisted of the entire disc organ, including the vertebral endplates (VEP), annulus fibrous (AF), and nucleus pulposus (NP) with adjacent vertebral bodies (VB).

All samples were placed in a custom-made apparatus for the spinal motion segment that we developed (S1 Fig); a total of 48 IVD motion segments were maintained in the apparatuses without loading (controls). The other 48 samples were kept in the apparatuses under a constant compressive load (0.5 MPa). Sixteen fresh samples were used for evaluation. All apparatuses were maintained in an incubator under standard culture conditions (37°C, 5% CO_2_).

The specimens were maintained in Dulbecco’s Modified Eagle’s medium (DMEM) supplemented with 20% fetal bovine serum (FBS; Invitrogen Life Technologies, Carlsbad, CA, USA), 50 mg/mL L-ascorbate, 100 U/mL penicillin, 100 mg/mL streptomycin, and 2.5 mg/mL Fungizone. NaCl was added to the DMEM to raise the osmolarity to 410 mOsm/kg[15]. The media were changed every two days. On the day before culturing (Fresh) and on days 3, 7, and 14 in culture, the motion segment was used for morphological and immunohistochemical evaluation, and the cranial VEP were used for ELISA, Western blotting and Real-time PCR. The dissection procedure of cranial VEP was as follows. First, the motion segment was horizontally incised through the disc (L4/5), the annulus fibrosus was cut with a blade, and the nucleus pulposus were excised using a micro curette. Then the VEP was reserved by removing the VB from the BEP under a microscope. See S2 Fig for a schematic of the procedure.

#### 1.2.2 Loading and culturing apparatuses used with the intervertebral disc motion segments

The apparatuses were designed for *ex vivo* culturing of IVD motion segments while simultaneously providing static axial compression. Each apparatus had two components: a loading frame and a culture chamber.

The loading frame provided a static load via weights on a mobile loading plate that could be moved along the optical axis. Because compression can cause the IVDs to change slightly in height, the mobile weight ensured that the full load would be applied to the specimen consistently. A gland was fixed to the bottom of the loading plate, through which the weight was applied to the model. A gland also covered the top of the chamber to ensure that the culture environment was closed (S3 Fig).

A disposable silicone tube was inserted into the bottom of the culture chamber. The tube functioned as an outlet through which the medium could be changed; the other end of the tube was plugged. The medium was changed with a syringe, and the manipulations were performed on an aseptic operation table. The tubing and needle were changed with each use to ensure sterility (S3 Fig).

Based on the characteristics of the spinal motion segment, the device was arranged on the top and base pedestals. Three jackscrews were inserted through the pedestal to fix one side of the VBs, allowing the motion segment to remain in an upright state so that the compression was always vertically loaded on the surfaces of the IVDs (S3 Fig).

All of the components of the device could be removed individually, and the materials were resistant to deformation and decomposition by disinfection treatments (e.g., high temperature and high pressure). The whole apparatus was small enough that multiple loading stations could be placed in the incubator at the same time (S1 Fig; patent number: ZL 201410513493.9).

In the previous experiment, the apparatuses were used to compare the effect of different loads (50 N, 100 N, 300 N, 500 N) on the NP of the rabbit IVD motion segment in organ culture[31]. The results showed that the internal pressure of the IVD was 0.55±0.12 Mpa under 300 N, and the levels of proteoglycan and collagen in NP decreased significantly under the constant static compression of this pressure load. However, we found that the static compression stimulated the synthesis of ECM within a brief period, and the activity and metabolic ability of NP can be maintained for 14 days[32]. In this study, we used a constant static compression under 300 N as the load.

#### 1.2.3 Evaluation of vertebral endplate morphology

The motion segments (n = 4) were taken from each group at each time point and were fixed in 10% formalin overnight. Decalcification in 19% EDTA up to 21 days was followed by the standard tissue processing method before paraffin embedding[30, 33]. Midsagittal sections were made at 5 μm intervals for hematoxylin and eosin staining (H&E staining). Histomorphological changes of IVD with cranial VEPs were evaluated after the slides were mounted.

#### 1.2.4 Enzyme-linked immunosorbent assay (ELISA)

The VEPs (n = 4 per group of per time point) were collected and washed with PBS and then shredded, homogenized, centrifuged at 3,000 r/min for 10 min at 4°C. The supernatant (1 mL) was transferred to a sample tube. According to instructions in the ELISA kit (Sigma, St. Louis, MO, USA, RAB0509), the standard was diluted in the dilution buffer at established ratios and mixed thoroughly. The wells of the ELISA plate were sequentially numbered and coated with 100 μl of capture antibody. Then, 50 μl of standard solution or sample was added to the designated wells, mixed thoroughly, and covered for incubation at room temperature for 2 h. The solution in each well was subsequently discarded, and each well was washed four times with washing buffer. Next, 200 μl of the corresponding enzyme-linked secondary antibody was added to each well, and the mixture was covered and incubated for 2 h at room temperature. The solution in each well was discarded, and each well was washed four times with washing buffer. Then, 200 μl of the chromogenic substrate was added to each well in the dark, and the solution was mixed and incubated for 30 min, after which 50 μl of stop solution was added and mixed. The absorbance value D of each well was measured using a microplate reader at 550 nm within 30 min after adding the stop solution. The samples were run in triplicate to ensure reproducibility of the results, and a standard curve was plotted to calculate the content of VEGFA and VEGFR2.

#### 1.2.5 Western blotting

The VEPs (n = 4 per group of per time point) were collected. An appropriate amount of lysis buffer was added, and each sample and was homogenized until fully cracked. After the lysate was centrifuged at 12,000 rpm/min for 15 min, all the protein solution in the upper layer was aspirated. The protein concentration was determined using the BCA method. The protein lysate was equally aliquoted and then mixed with loading buffer, boiled, and denatured for sodium dodecyl sulfate-polyacrylamide gel electrophoresis (SDS-PAGE). The following steps were then performed: membrane transfer, 5% BSA blocking for 1 h, overnight incubation with the corresponding VEGFA antibody (Abcam, Cambridge, UK, AB32152, diluted 1:1,000), Tris-buffered Saline and Tween (TBST) wash, incubation with secondary antibody (Abcam, Cambridge, UK, AB205718, diluted 1:2,000) for 1 h, TBST wash and incubation with an ECL reagent to detect the level of protein expression. Grey values were determined using a computer-assisted image analyzer.

#### 1.2.6 Real-time PCR

The VEPs (n = 4 per group) were collected at each time point. Trizol reagent (Invitrogen Life Technologies, Carlsbad, CA, US) was used following the manufacturer’s instructions. The mixtures were placed on ice for sonication at 30% amplitude. Each sample was sonicated for 3 s and then paused for 2 s for a total sonication time of approximately 30 s. Subsequently, chloroform, isopropanol, and alcohol were added one by one to the samples for total RNA extraction. The RNA concentration was then determined. Total RNA was reverse-transcribed into cDNA using a reverse transcription (RT) kit. PCR amplification was performed using the first-strand cDNA as the template. The PCR reaction conditions were as follows: pre-denaturation at 94°C for 5 min, followed by 32 cycles of denaturation at 94°C for 20 s, annealing at 58°C for 20 s, and extension at 72°C for 20 s, followed by a final extension at 72°C for 10 min. The PCR products were separated with agarose gel electrophoresis. Primers for the rabbit GAPDH and VEGFA genes were custom designed using Primer 5.0 software (Applied Biosystems, Foster City, CA, USA) [Table 1]. Grey values and relative intensity were determined and calculated using a gel imaging system.

**Table 1:** Details of the primers for the target and reference genes used in quantitative real-time PCR.

Gene	Primers	Primer Sequence
***GAPDH***	tuzi-GAPDH-F (5′–3′)	GCTGAGAATCTGCCCCTCTT
	tuzi-GAPDH-R (5′–3′)	TTTCATGACAAGGTAGGGCTCC
***Tu-VEGFA***	Tu-VEGFA-F (5′–3′)	CCTTCACCTACTGTCATCTGCTTCC
	Tu-VEGFA-R (5′–3′)	GCTACTACTTCGTCCACTCTTCTTCC

***GAPDH*:** glyceraldehyde-3-phosphate dehydrogenase; ***VEGFA***: vascular endothelial growth factor; F: Forward; R: Reverse.

#### 1.2.7 Immunohistochemistry

The sagittal sections (n = 4 per group) at each time point were dewaxed, hydrated, and washed using routine methods[34, 35]. Immunohistochemistry was performed for MVD, Aggrecan, COL2a1, and VEGFA of the vertebral endplate. Antigen retrieval was performed using a heat-induced antigen retrieval protocol. The sections were blocked with normal goat serum for 5 min. The disc sections were incubated with primary antibodies against CD34 (GeneTex, Irvine, CA, USA, GTX28158, diluted 1:500), Aggrecan (Novus, NB120-11570, diluted 1:200), collagen Type II alpha 1 (Abcam, ab34712, diluted 1:200), and VEGFA (Abcam, Cambridge, UK, AB32152, diluted 1:250) overnight at 4°C. This was followed by incubation with corresponding HRP-conjugated secondary antibodies for 50 min at 37°C. The tissue sections were developed with 3,3'-diaminobenzidine(DAB) and were lightly counterstained with hematoxylin, dehydrated, and mounted onto slides. Negative controls were incubated in nonimmune rabbit or mouse serum replaced the primary antibody and were processed in the same way as the experimental sections.

The vascular buds were statistically counted using the microvessel density (MVD) method described by Weidner[36]. Briefly, the number of microvessels in high fields was determined, and the number of microvessels in five fields was recorded. All single endothelial cells and endothelial cell clusters stained brown were taken as one vessel, and the mean value was taken as the MVD of the section. Specifically, the five densest fields of microvessels at the certain depth[37] underneath the endplate were identified with low field scanning first, and then the stained microvessels were counted in the range of high field. The mean number of microvessels in the section was taken as the mean of the five field counts, and the mean number of microvessels in each section was taken as the number of microvessels for that specimen.

Semi quantification of immunopositivity was also calculated for Agg, COL2a1, and VEGFA. Briefly, the staining of images was obtained under an optical microscope (Nikon TE2000). Five randomly selected brown-yellow staining regions of interest (ROI) in each image were captured for analysis with Image-Pro Plus 6.0 software (Media Cybernetics Inc, Bethesda Maryland, USA) to measure the sum of integrated optical density (IOD), using the method introduced by Xavier[38]. The optical density was calibrated, and the image was converted to a grayscale image; the values were counted.

#### 1.2.8 Statistical methods

All data are represented as the mean values (s) ± standard deviation (s) of individual analysis. The difference between control and static load groups at each time point was calculated using the unpaired student’s *t*-test. The data collected at different time points in the same group were assessed for normality and equal variances, after which two-way ANOVA and Tukey’s posthoc tests were performed. The data were analyzed with SPSS software (version 16.0, IBM, Chicago, IL, USA). Statistical significance was set at P < 0.05.

## Results

### 2.1 Histomorphological characteristics of the degenerating IVDs

**Fig 1:**
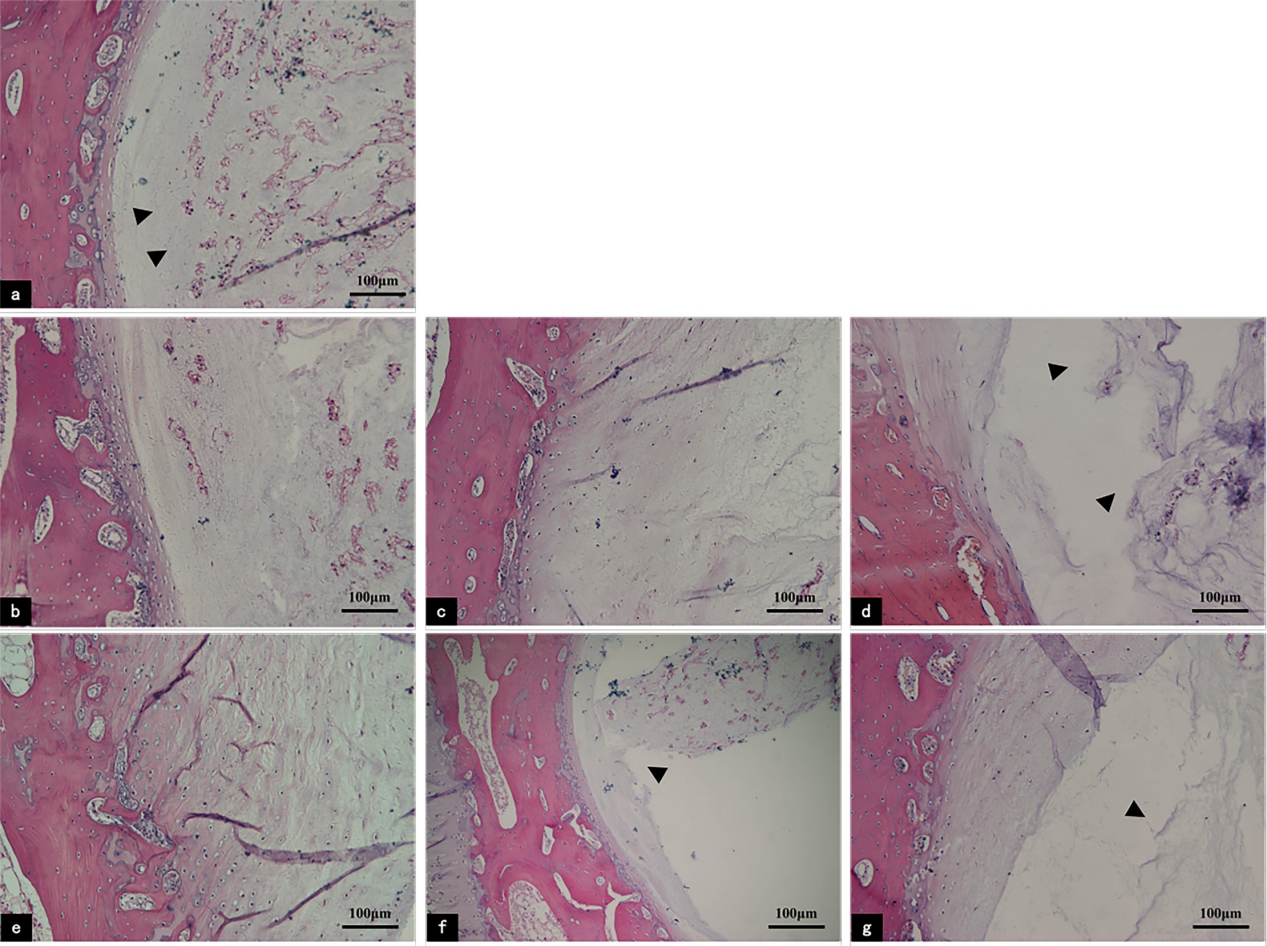
H&E staining of the sagittal sections of IVD in the control and pressure groups. (a) Fresh tissue; (b, c, and d) tissue histological observations of organ controls on days 3, 7, and 14; (e, f, and g) histological observations of tissues under static load on days 3, 7, and 14. Fresh samples show that the structure of the VEP was intact (a, arrowhead). After one week of culture, the integrity of the IVDs in the control group was good; however,r under constant compression, the separation of IVDs integrity appeared (f, arrowhead), and the matrix was lightly stained. In the two groups, culturing for two weeks led to the loss of the tight architecture of the IVDs (d and g, arrowheads); these changes were more obvious under constant compression than in the controls. **IVD:** intervertebral disc; **NP:** nucleus pulposus; **VEP:** vertebral Endplate.

As shown by H&E staining, the IVDs were structurally intact in the fresh sample, the nucleus pulposus (NP) was tightly organized with the vertebral endplate (VEP), and there were abundant cells in the VEP matrix. The cells were large and tightly aligned, and they contained many neatly arranged collagen fibre bundles. In addition, vascular buds were visible in the cranial VEPs (Fig 1A). After one week of culture, the integrity of the IVDs in the control group was still maintained, although the cells and the matrix were lightly stained in the VEP (Fig 1C). Under the static load, the integrity of the tissue was maintained for three days (Fig 1E). After culturing for seven days, the separation of the IVDs integrity appeared and the cells gradually dispersed, with a decreased number and reduced size. The vascular buds were significantly fewer. The cytoplasm and nuclei of the remaining cells were smaller than those of the normal cells, and they also had a disorderly arrangement of collagen fibres. In addition, the VEP matrix was lightly stained (Fig 1F). Culturing for two weeks led to the loss of the tight architecture of the IVDs in the two groups (Fig 1D–1G), and there were significantly fewer vascular buds; however, these changes were more readily visible under constant compression than unloaded conditions.

### 2.2 The concentration of VEGFA and VEGFR2 in the vertebral endplates

**Fig 2:**
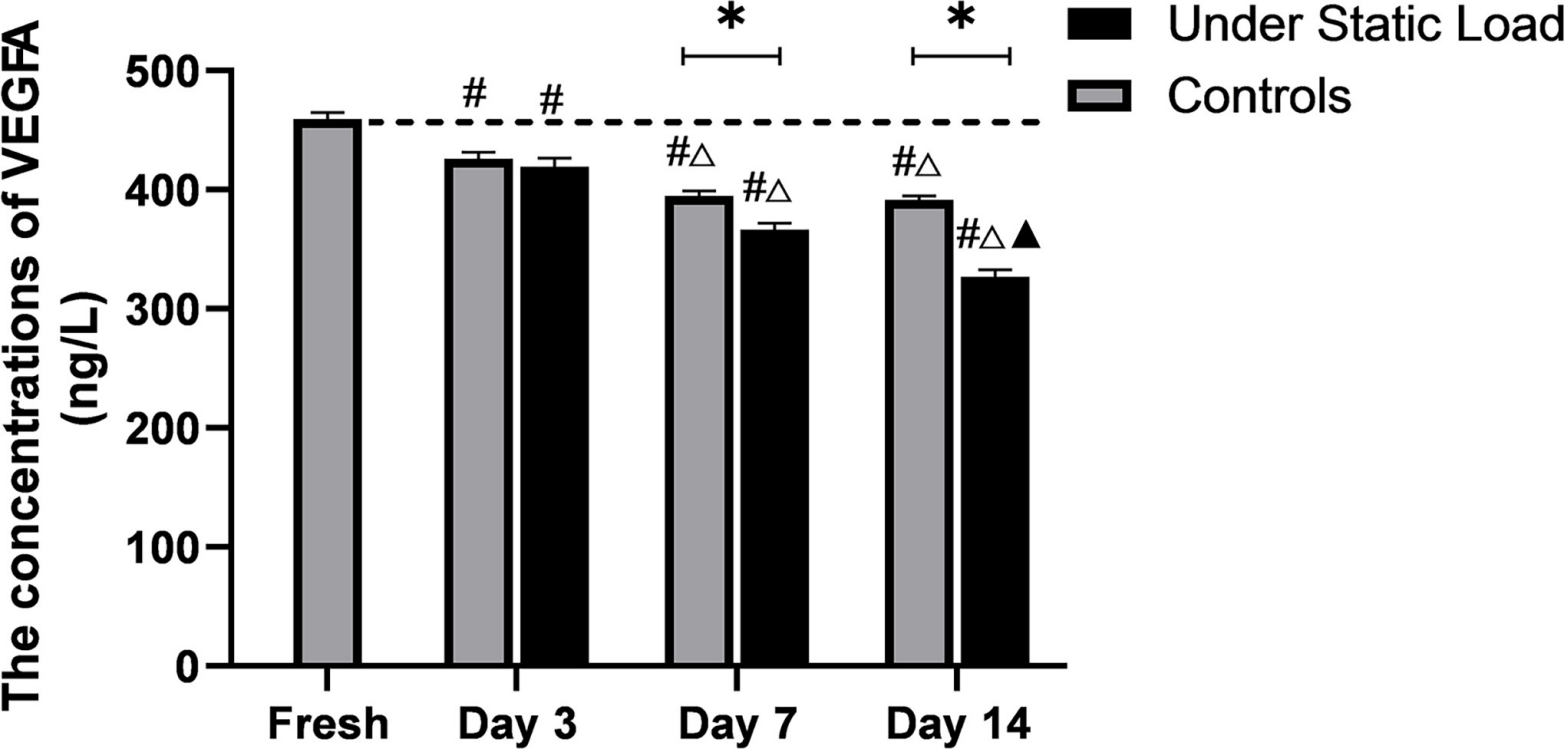
The concentrations of VEGFA secreted from the vertebral endplate during organ culture (ng/L, n = 4, x ± s). (*P<0.05 vs Controls; ***#*** *P* < 0.05 vs Fresh; △*P* < 0.05 vs Day 3; ▲*P* < 0.05 vs Day 7).

The concentration of VEGFA in the cranial VEPs tissues was determined by ELISA for each group at each time point. As shown in Fig 2, the VEGFA concentration in the vertebral endplate tissues was decreased in controls compared to the fresh tissue samples, and the concentration continued to decrease after one week of culture. There was no significant difference between the samples that were cultured for one week and two weeks. Under static load, the VEGFA concentration was decreased at three days compared with the fresh samples, but there was no significant difference when compared with the controls. After one week of culture, the VEGFA concentration decreased and was significantly different from the controls. The concentration of VEGFA was further reduced after two weeks of culture.

**Fig 3:**
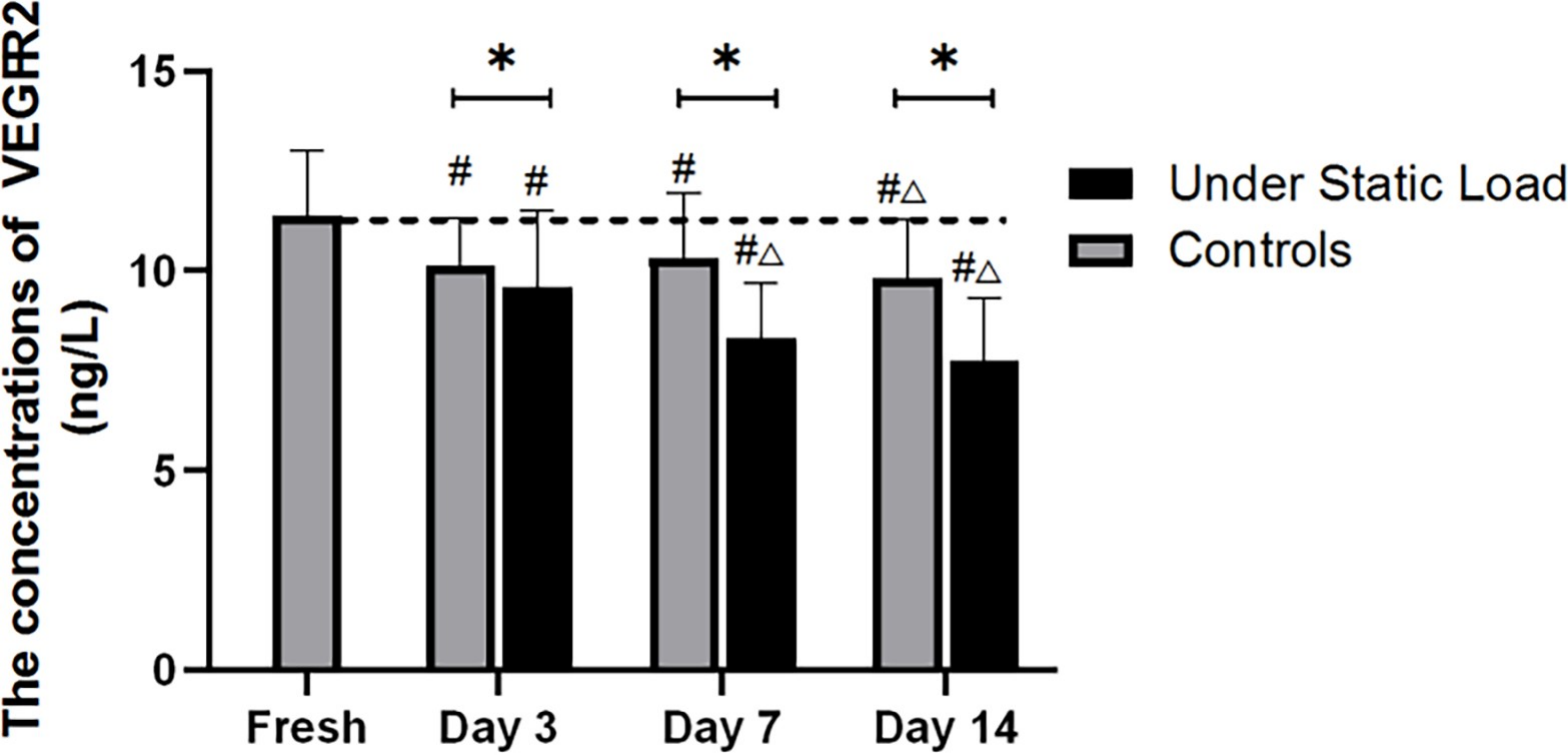
The concentrations of VEGFR2 secreted from the vertebral endplate during organ culture (ng/L, n = 4, x ± s). (*P<0.05 vs Controls; ***#*** *P*<0.05 vs Fresh; △*P*<0.05 vs Day 3; ▲*P*<0.05 vs Day 7).

As shown in Fig 3, the concentration of VEGFR2 was decreased in the two groups after culture compared with the samples before culture. The concentration of VEGFR2 was significantly decreased in the pressure group compared to the control group. There was no significant change in the VEGFR2 concentration in the control group within one week of culture, but the concentration was significantly decreased during the second week of culture. In the pressure group, the VEGFA concentration in the samples was significantly lower after seven days of culture than on day three. There was no significant difference after one week of culture.

### 2.3 Detection of VEGFA expression in the vertebral endplates by Western blot

**Fig 4:**
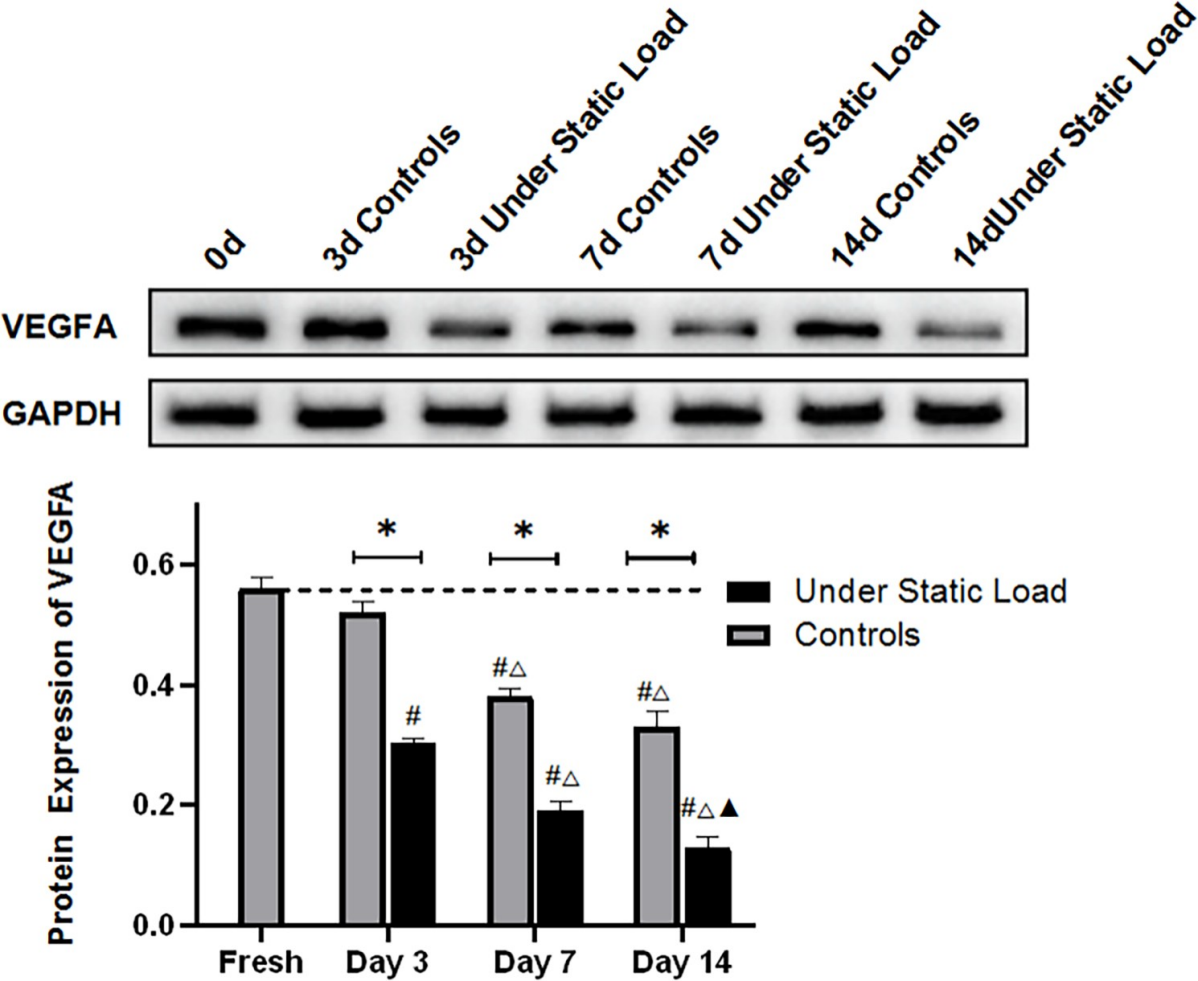
VEGFA protein expression level in the vertebral endplates was determined using Western blot analysis (n = 4). (*P<0.05 vs Controls; **#** P<0.05 vs Fresh; △P<0.05 vs Day 3; ▲P<0.05 vs Day 7).

As shown in Fig 4, the Western blot results showed that the constant compressive load caused a significant decrease in the VEGFA expression level in the vertebral endplates compared with the fresh samples and the controls. With the prolongation of culture, the expression gradually decreased and was significantly different from that of the controls during the culture period.

### 2.4 Detection of VEGFA expression in the vertebral endplates by real-time PCR

**Fig 5:**
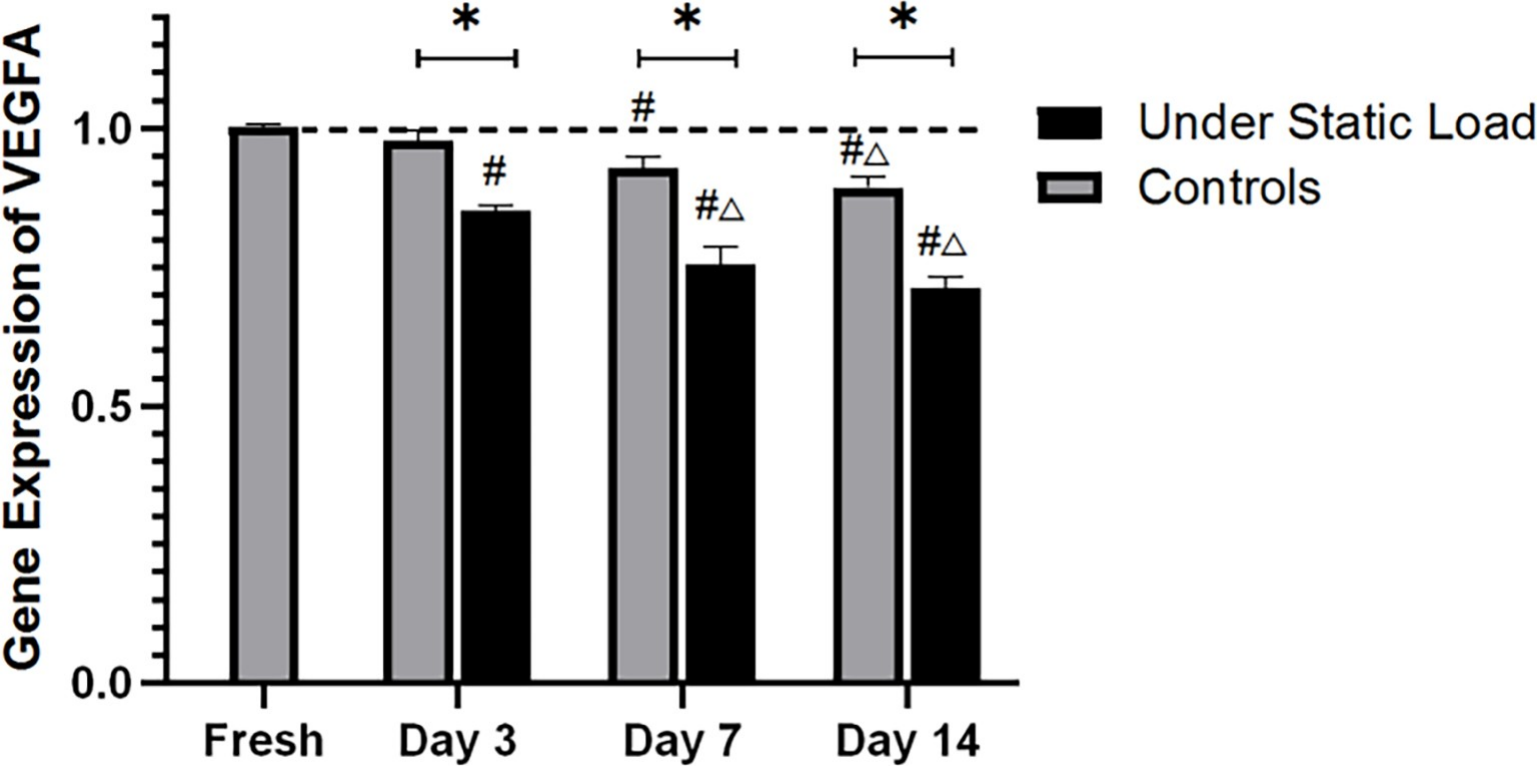
VEGFA gene expression quantified by real-time PCR during organ culture (n = 4). (*P<0.05 vs Controls; **#** P<0.05 vs Fresh; △P<0.05 vs Day 3).

RT-PCR was performed to detect the expression of the VEGFA gene (S1 and S2 Supporting Information). As shown in Fig 5, compared with the fresh samples, the expression of VEGFA was not significantly decreased in the controls at day three of culture. At day seven, the VEGFA expression level was lower than that in the fresh samples. There was no difference in expression level between day 14 and day seven, but both differed significantly from levels recorded on day three. The VEGFA expression was significantly decreased under the compressive load on day three. After seven days of culture, the expression decreased further, but there was no significant difference between day seven and day 14. At each time point, the expression levels of VEGFA under the constant compressive load were all significantly different from those of the controls.

### 2.5 Number of vascular buds in the vertebral endplates

**Fig 6:**
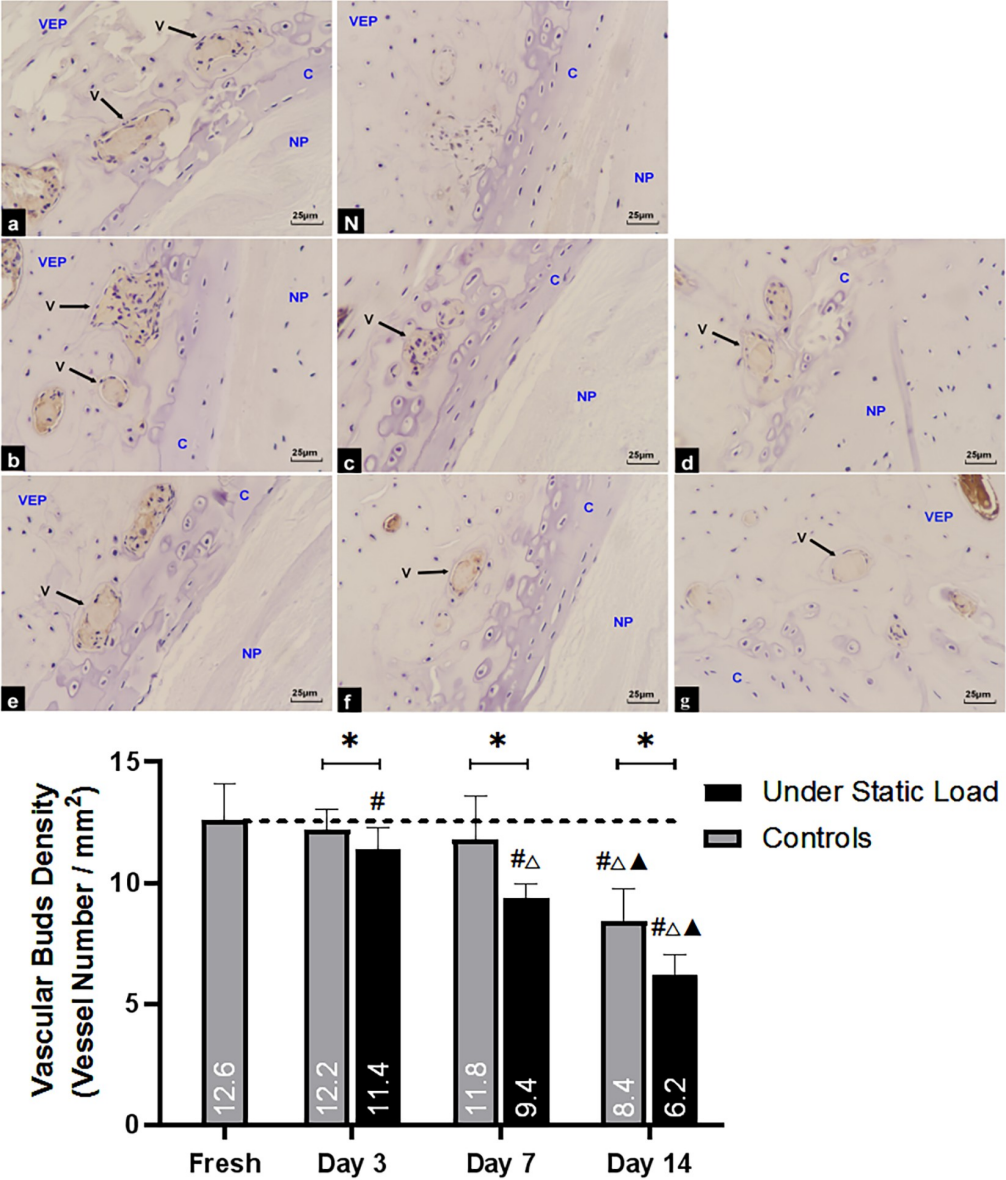
Numbers of vascular buds in vertebral endplates of the two groups (n = 4). (a) Fresh tissue; (N) Negative control; (b, c, and d) Controls on days 3, 7, and 14, respectively; (e, f, and g) Under constant compression on days 3, 7, and 14, respectively. NP = nucleus pulposus, VEP = vertebral endplate, C = cartilage, V = vascular buds. (*P<0.05 vs Controls; **#** P<0.05 vs Fresh; △P<0.05 vs Day 3; ▲P<0.05 vs Day 7).

Compared with the fresh samples (Fig 6A), the number of vascular buds in the cranial VEPs in the controls was not significantly decreased after three days of culture (Fig 6B), and the density of the vascular buds was slightly reduced after one week of culture (Fig 6C). There were significantly fewer vascular buds in the VEPs after three days of culture under the constant compression compared to the fresh samples (Fig 6E); this difference became more pronounced after one week (Fig 6F). The constant compressive load condition was associated with significantly fewer vascular buds than in the nonloaded control group at each point in time during the culture.

### 2.6 Detection in the vertebral endplates by immunohistochemistry

#### COL2a1

**Fig 7:**
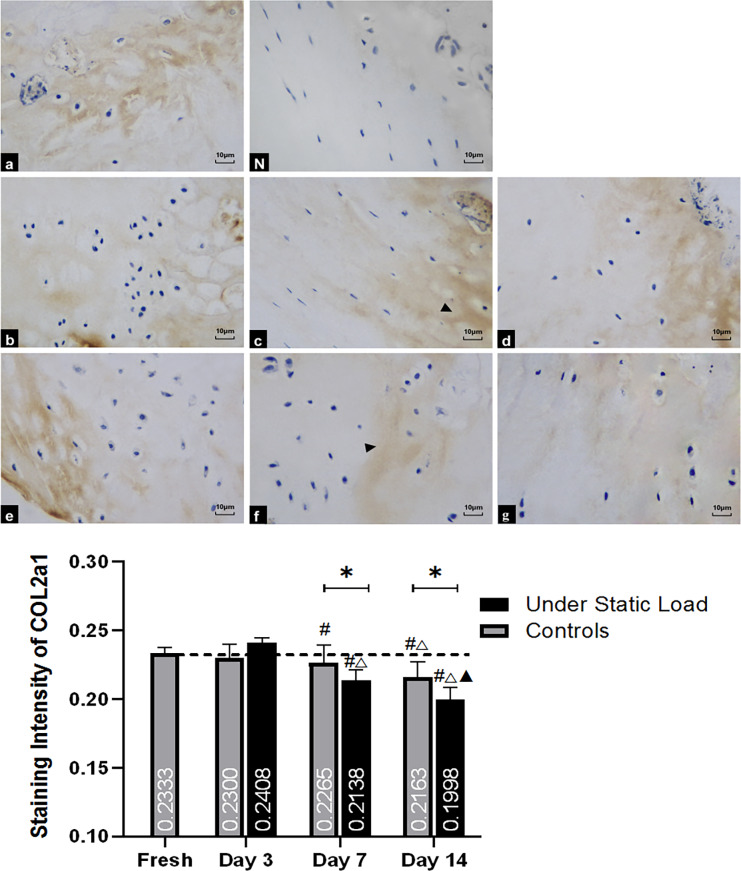
Immunohistochemical staining of COL2a1 in the vertebral endplate (n = 4). (a) Fresh tissue; (N) Negative control; (b, c, and d) Controls on days 3, 7, and 14, respectively; (e, f, and g) Under constant compression on days 3, 7, and 14, respectively. The intensity in the cartilage of VEPs showed no significant difference between the two groups with fresh tissues during the first three days. However, after seven days of culture, the staining was visibly reduced in both groups, and the samples under constant compression showed more pronounced decreases than the controls (c and f, arrowhead). By day 14, the staining had decreased further (d and g, arrowhead). VEP = vertebral endplate. (*P<0.05 vs Controls; **#** P<0.05 vs Fresh; △P<0.05 vs Day 3; ▲P<0.05 vs Day 7).

The staining intensity of collagen Type II alpha 1 in the cartilage of VEPs showed that there was no significant difference between the two groups with fresh tissues during the initial three days (Fig 7A, 7B and 7E). However, after seven days of culture, the staining was reduced in both groups (Fig 7C and 7D), and the samples under the static load decreased more significantly compared with the controls. By day 14, the staining was further decreased and significantly different from that observed in the controls (Fig 7D–7G).

#### Agg

**Fig 8:**
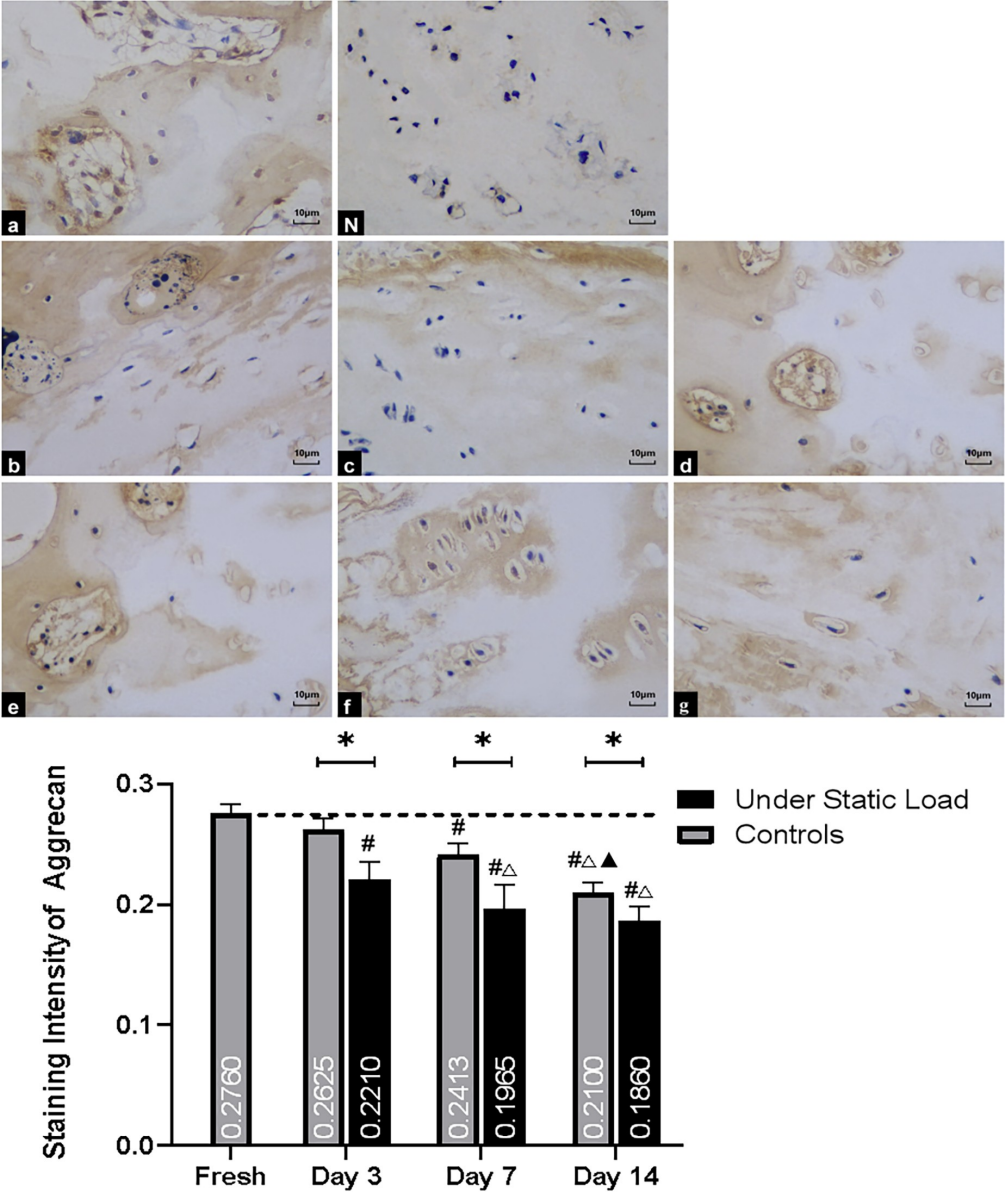
Immunohistochemical staining of Agg in the vertebral endplate (n = 4). (a) Fresh tissue; (N) Negative control; (b, c, and d) Controls at days 3, 7, and 14, respectively; (e, f, and g) Under constant compression at days 3, 7, and 14, respectively. The staining intensity in the cartilage of VEPs was significantly lighter in the pressure group than the controls and fresh samples on day three. After one week of culture, the staining intensity in the two groups was markedly lower than in fresh samples. After two weeks of culture, the staining intensity was lightened further in both groups. VEP = vertebral endplate. (*P<0.05 vs Controls; **#** P<0.05 vs Fresh; △P<0.05 vs Day 3; ▲P<0.05 vs Day 7).

The aggrecan staining in the cartilage of VEPs was significantly lighter under the static load condition than that of controls and fresh samples (Fig 8). Moreover, there was a significant difference between the two groups. After one week of culture, the staining intensity in both groups was visibly lower than in fresh samples, and the staining decreased to a greater extent in the pressure group compared to the control group. After two weeks of culture, the staining lightened further in controls, but there was no significant decrease in intensity in the pressure group over seven days. There were still differences between the two groups.

#### VEGFA

**Fig 9:**
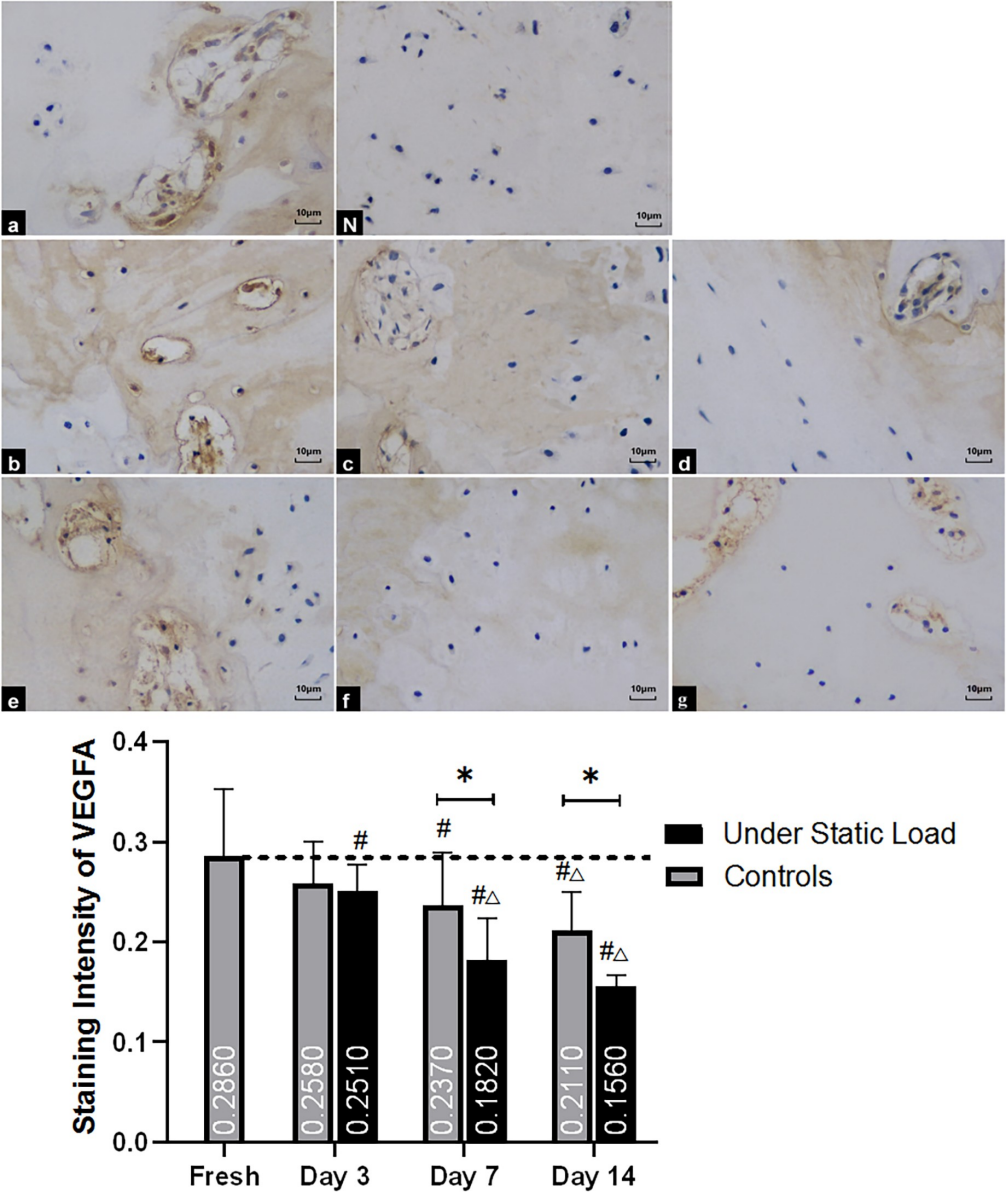
Immunohistochemical staining of VEGFA in the vertebral endplate (n = 4). (a) Fresh tissue; (N) Negative control; (b, c, and d) Controls at days 3, 7, and 14, respectively; (e, f, and g) Under constant compression at days 3, 7, and 14, respectively. The extracellular matrix in the cartilage of VEPs was stained brown-yellow in fresh tissue. The staining was slightly reduced in two groups, but there was no significant difference between them; however, the staining was reduced after seven days for both groups, and the samples under constant compression were significantly different from the controls. After two weeks, the staining of the matrix became lighter in the control group and noticeably lighter in the pressure group. VEP = vertebral endplate. (*P<0.05 vs Controls; **#** P<0.05 vs Fresh; △P<0.05 vs Day 3; ▲P<0.05 vs Day 7).

Before culture, anti-VEGFA immunohistochemistry in the cartilage of VEPs showed that the extracellular matrix was stained brown-yellow and had a dark color (Fig 9); the staining of the tissue was less intense than that of the fresh samples after three days of culture under the compressive load. However, there was no significant difference in staining compared with the controls. After one week of culture, the staining intensity was reduced, and there were significant differences when compared with the controls. After two weeks of culture, the staining intensity was lightened further, and there were still differences between the two groups.

## Discussion

We used an *ex vivo* rabbit IVD model to study constant compressive loading and its effects on cranial vertebral endplate (VEP) vascularity and degradation. Results of HE, ELISA, Western blot, RT-PCR, and immunohistochemistry assays showed that the integrity of the morphology of the VEPs was gradually destroyed, and the levels of proteoglycan and collagen II decreased significantly. During the disc degeneration process, which was caused by constant compression, the number of vascular buds in the VEPs gradually decreased from the early stage of culture (3 d). The expression of VEGFA and its receptor in the VEPs also decreased. These results suggest a correlation between vascular bud lesions and decreases in VEGFA expression in the presence of IDD induced by constant compression.

Mechanical load is the main function of the IVD, and abnormal load is an important initiator of IDD[39, 40], whereas static load is a typical type of abnormal loading for IVD tissues[41]. Previously, we observed the effects of constant compression on the nucleus pulposus tissue. The results showed that a short-term compression is good for preventing the expansion of IVD tissues and maintaining cell viability, but that a constant static load will gradually inhibit the metabolic activities of the nucleus pulposus cells, leading to significant degeneration of the nucleus pulposus[32]. When the spine is under abnormal loading, the proteoglycan content in the VEP will change, promoting its calcification and reducing its elasticity, in addition to weakening the abilities of the spine to absorb shock and evenly transmit weight[42]. Furthermore, the accumulation of metabolites in the IVD can activate catabolic enzymes, causing matrix destruction, cellular metabolic disorders, and cell death[43, 44]. Relevant studies[11–13] have also confirmed that an abnormal load can slow the blood flow inside the bony endplate and lead to blood stasis, thereby causing injury to vascular endothelial cells and thrombosis. These changes result in a progressive reduction in the distribution of vascular buds within the VEP and trigger nutritional disorders in the IVD. However, there has been no in-depth study of the mechanism underlying vascular bud lesions caused by an abnormal load.

Studies have shown that VEGF is closely related to vascularization inside the IVD[16, 20, 33]. VEGF promotes angiogenesis, endothelial cell proliferation, and migration, and also enhances vascular permeability[45]. In the clinic, Wang and Xu *et al*.[15, 46] found that VEGF expression is present in normal human endplates and is significantly higher than the expression in the degenerative endplates. Moreover, Xu[47] constructed a pcDNA3.1-VEGFA165 plasmid and directly injected it into rabbits with degenerative IVD, suggesting that VEGF can increase the number and diameter of vascular buds and delay the calcification of VEPs and IDD. There are various types of cells in the VEP[48], and chondrocytes are the major cell type in endplates. Studies[49, 50] have shown that chondrocytes could improve the cartilage tissue degeneration by paracrine VEGF. Therefore, the VEGF secreted by these chondrocytes in VEP may also be related to vascular bud function in the endplate and endplate degeneration. The above in-vivo studies showed IDD to be related to abnormal load and the changes in VEGF of the endplate, and that the mechanical load can regulate the expression of VEGF in various cell types *in vivo*[51]. However, the effect of an abnormal load on VEGF in the VEP is not apparent. For these reasons, we used VEGFA expression to explore the molecular mechanism underlying vascular bud injury in the VEP caused by a constant compressive load.

To achieve our objectives, we used an *ex vivo* culture model. Based on the *ex vivo* abnormal loading model, the whole IVD organ, together with the vertebral bodies (VBs), was used for the *ex vivo* whole-organ culture, thus retaining the important inter-cellular and matrix-matrix interactions inside the IVD. As such, this *ex vivo* model is similar to *in vitro* intervertebral disc organ culture models without endplate and VBs[19, 22, 52]. Additionally, because the *in vivo* mechanical environment is complex, the load parameters cannot be controlled entirely. In this *ex vivo* model, the weight can be simply applied to IVDs[53]; thus, this model is more suitable for observation of the influence of specific mechanical factors on IVD than other models[23, 24, 54]. Unlike in large animals, IDD progresses rapidly in small animals[53, 55], making them more adequate to the study of the cellular metabolic function of IVD. The surface ratio and diffusion distance of the rabbit IVD attached to the endplate surface are suitable for the exchange of nutrients and metabolites[37]. Accordingly, the rabbit spinal motion segment was selected as a model in the current mechanobiological study covering the nutrient transmission function of the endplate. Our study results suggest that VEGFA is involved in the process of abnormal load-induced IDD and that vascular bud lesions are related to a decreased expression of VEGFA.

The current study has several limitations that require discussion. First, the constant compressive load resulted in a lack of fluid flow in this model. *In vivo* studies have shown a clear correlation between VEGF expression and blood flow[56–58]. However, it is not clear whether the VEGF expression decrease in this study is directly caused by a static load or due to the lack of fluid flow in the static mechanical state. Related *in vivo* and *ex vivo* studies[23, 24, 59, 60] have shown that dynamic axial loading can promote the infiltration of the endplate, enhance the transport of internal nutrients and metabolites, and maintain the biosynthetic activity of the IVD. In another study, VEGF and a periodic compressive load were found to promote vessel growth in the cartilage and accelerate tissue repair[61, 62]. We speculate that dynamic loading is also related to the regulation of the expression of VEGF and improving the condition of vascular buds in the VEPs. In future work, we plan to study the effect of dynamic loading on VEGF in the *ex vivo* model to establish the relationship between fluid flow and VEGF expression. To further establish the relationship between the VEGF and IDD in the static load state, we plan to add to the culture medium biologically active reagents, such as VEGFA inhibitors and activators, to observe changes in IDD. We hope to provide new therapeutic targets and strategies for the effective prevention and treatment of IDD from the perspective of mechanics and nutrition supply using the mechanistic techniques described above.

Second, other studies have shown that VEGF can be detected in both clinical cases[16] and animal models[47] of IDD, and is positively correlated with the level of vascular invasion and the extent of degeneration. Therefore, some researchers believe that high VEGF expression in the IVD marks the beginning of IDD and can promote and induce IDD[63]. However, the above studies chose nucleus pulposus tissue as the research object. When the nucleus pulposus protrudes into the vertebral body or the endplate degenerates and loses its protective function, the nucleus pulposus is in direct contact with blood vessels. Although this contact increases the substance exchange rate, blood vessels may grow into the nucleus pulposus and aggravate inflammation and pain symptoms because of the effects of VEGF[64]. Therefore, high VEGF expression in the nucleus pulposus tissue often indicates the occurrence of inflammation and degeneration. However, if the endplate is used as the research object, regulating the VEGFA expression in the endplate chondrocytes may improve the conditions of the lesions to the vascular buds in the VEPs, thereby enhancing the nutritional supply function in IVD and thus playing a role in delaying IDD. At present, the details of the mechanism underlying the functions of VEGF in IDD remain unclear. The relevant factors affecting the cells of VEP have complex interrelationships in the IVD microenvironment of the endplate-nucleus pulposus, and VEGF may be one of the key points of action[47, 65]. We will continue to study the mechanisms of VEGF activation, as they are closely related to vascular bud regulation. We will also study the relationship between VEGF and other cytokines.

Third, the *ex vivo* model used here has several limitations. In addition to the mechanical load, the conditions of oxygen, glucose, pH, osmotic pressure, growth factors, and other factors in the culture system can affect the cell phenotype and metabolism in an *ex vivo* culture model, and the determination of a narrow optimum range for each of these parameters requires a large number of experiments. Furthermore, notochord cells persisted in the rabbit IVD, and these may have promoted the proliferation of pulposus cells in the nucleus and the synthesis of the extracellular matrix such as proteoglycans[66]. However, Guehring[67, 68] found signs of decreased endplate cellularity and increased endplate sclerosis and fibrosis after loading in an *in vivo* rabbit IDD model. These experiments show that notochord cells were less resistant to mechanical stress caused by limited nutrition through structurally altered endplates after increased intradiscal pressure. The underlying influence of notochord cells on the IVD under abnormal load conditions is not fully understood. We used the same *ex vivo* degenerative model as in a previous study[32]. Results showed that a static load could stimulate the synthesis of type II collagen in the nucleus pulposus matrix during the first three days, but there was no such phenomenon in the VEP matrix in this experiment. Whether this observation is related to the intolerance of notochord cells to the load needs further study. Moreover, the effect of VBs on the viability and function of IVD should be addressed. Some researchers[52, 69] have argued that the VB can prevent nutrients from diffusing from the VEP to the inside of the disc, resulting in significant IDD during culture in the *ex vivo* model. However, studies reported by Lim[70] and Seol[71] used *in vitro* cultured small animal spinal motion segments showed that even retained VBs did not affect the diffusion of nutrients. By observing the diffusion of nutrients, Dong Wang[28] concluded that VBs were preserved in small animals, and the viability of IVD can be maintained because of the small distance required for diffusion of nutrients into the disc. Lee[21] also maintained that the surface ratios of IVDs from rats and rabbits are smaller than those of humans, so the diffusional limitation in culturing large IVDs as humans and cattle would be minimized in studies performed in rats and rabbits. We share the view of Lim[70], in that the *ex vivo* culture of spinal motion segments provides a physical environment relatively similar to that of a living animal, and the adjacent VBs facilitated the use and control of mechanical loads. In this way, the rabbit spinal motion segment is useful for assessing the influence of specific mechanical factors on IVD.

In conclusion, constant compression caused degeneration of the vertebral endplate and decreased vascular buds in the endplate, thereby accelerating disc degeneration; VEGFA is involved in this process. We anticipate that regulating the expression of VEGFA may improve the condition of the lesions to the vascular buds in the VEPs, thus enhancing the nutritional supply function in IVD and providing new therapeutic targets and strategies for the effective prevention and treatment of IDD.

## Supporting information

S1 Fig View of the specimen culturing conditions. The specimens were maintained in custom-made apparatuses without loading or under a constant compressive load (a); the apparatuses were situated inside a 37°C incubator with 5% CO_2_ and 100% humidity (b).
(TIF)

S2 Fig Dissection procedure of the cranial vertebral endplate. VB: vertebral bone, AF: annulus fibrosus, NP: nucleus pulposus, VEP: vertebral endplate, BEP: bony endplate, CEP: cartilage endplate.
(TIF)

S3 Fig Loading and organ culturing apparatuses for the rabbit IVD motion segments (diagrammatic sketch). 1. Weight; 2. Loading plate; 3. Gland; 4. Optical axis; 5. Chamber; 6. Chamber fixed base; 7. Outlet; 8. Top pedestal; 9. Fluid level observation hole; 10. IVD motion segments; 11. Base pedestal; 12. Jackscrews.
(TIF)

## References

[1] Stover JD, Farhang N, Berrett KC, Gertz J, Lawrence B, Bowles RD. CRISPR Epigenome Editing of AKAP150 in DRG Neurons Abolishes Degenerative IVD-Induced Neuronal Activation. Molecular Therapy the Journal of the American Society of Gene Therapy. 2017. 10.1016/j.ymthe.2017.06.010 PMC5589089 28676344

[2] Oki S, Matsuda Y, Shibata T, Okumura H, Desaki J. Morphologic differences of the vascular buds in the vertebral endplate: scanning electron microscopic study. Spine. 1996;21(2):174 10.1097/00007632-199601150-00003 8720400

[3] Kobayashi S, Baba H, Takeno K, Miyazaki T, Uchida K, Kokubo Y, et al Fine structure of cartilage canal and vascular buds in the rabbit vertebral endplate. Laboratory investigation. Journal of Neurosurgery Spine. 2008;9(1):96 10.3171/SPI/2008/9/7/096 18590419

[4] Ariga K, Miyamoto S, Nakase T, Okuda S, Meng W, Yonenobu K, et al The relationship between apoptosis of endplate chondrocytes and aging and degeneration of the intervertebral disc. Spine. 2001;26(22):2414–20. 10.1097/00007632-200111150-00004 11707702

[5] Shirazi-Adl A,., Taheri M,., Urban JPG. Analysis of cell viability in intervertebral disc: Effect of endplate permeability on cell population. Journal of biomechanics. 2010;43(7):1330–6. 10.1016/j.jbiomech.2010.01.023 20167323

[6] Nguyen C, Poiraudeau S, Rannou F. Vertebral subchondral bone. Osteoporosis International. 2012;23(s8):p.857–60. 10.1007/s00198-012-2164-x 23179569

[7] Yamaguchi T, Goto S, Nishigaki Y, Orías AAE, Bae WC, Masuda K, et al Microstructural analysis of three‐dimensional canal network in the rabbit lumbar vertebral endplate. Journal of Orthopaedic Research. 2015;33(2):270–6. 10.1002/jor.22759 25367593

[8] Benneker LM, Heini PF, Alini M, Anderson SE, Ito K. 2004 Young Investigator Award Winner: vertebral endplate marrow contact channel occlusions and intervertebral disc degeneration. Spine. 2005;30(2):167–73. 10.1097/01.brs.0000150833.93248.09 15644751

[9] Cao Y, Liao S, Zeng H, Ni S, Tintani F, Hao Y, et al 3D characterization of morphological changes in the intervertebral disc and endplate during aging: A propagation phase contrast synchrotron micro-tomography study. Scientific Reports. 2017;7:43094 10.1038/srep43094 28266560 PMC5339826

[10] Xu HM, Wang YL, Jin HM, Xu DL, Wang XY. A novel micro-CT-based method to monitor the morphology of blood vessels in the rabbit endplate. European Spine Journal. 2016;26(1):1–7. 10.1007/s00586-016-4886-5 27832363

[11] AG R, CK S, FL A, AE R-S, AJ B, S M, et al Human disc nucleus properties and vertebral endplate permeability. Spine. 2011;36(7):512–20. 10.1097/BRS.0b013e3181f72b94 21240044 PMC3062730

[12] Arun R, Freeman BJ, Scammell BE, Mcnally DS, Cox E, Gowland P. 2009 ISSLS Prize Winner: What influence does sustained mechanical load have on diffusion in the human intervertebral disc?: an in vivo study using serial postcontrast magnetic resonance imaging. Spine. 2009;34(21):2324–37. 10.1097/BRS.0b013e3181b4df92 19755934

[13] Hwan Tak H, Yon Jin C, Tan BHM, Tony S, Hee Kit W. Vascularization and morphological changes of the endplate after axial compression and distraction of the intervertebral disc. Spine. 2011;36(7):505–11. 10.1097/BRS.0b013e3181d32410 20975621

[14] Salo J, Kaigle HA, Indahl A, Mackiewicz Z, Sukura A, Holm S, et al Expression of vascular endothelial growth factor receptors coincide with blood vessel in-growth and reactive bone remodelling in experimental intervertebral disc degeneration. Clinical & Experimental Rheumatology. 2008;26(6):1018–26. 19210865

[15] Wang H, Chen XW, Hong-Guang XU, Ding GZ, Liu P, Huang DG, et al Correlation between VEGF and HIF-1α expression in vertebral cartilage endplate of cervical spondylosis patients. Journal of Practical Medicine. 2008.

[16] Han IB, Ropper AE, Teng YD, Shin DA, Jeon YJ, Park HM, et al Association between VEGF and eNOS gene polymorphisms and lumbar disc degeneration in a young Korean population. Genetics & Molecular Research. 2013;12(3):2294–305. 23884772 10.4238/2013.July.8.10

[17] Gantenbein B, Grunhagen T, Lee CR, van Donkelaar CC, Alini M, Ito K. An in vitro organ culturing system for intervertebral disc explants with vertebral endplates: a feasibility study with ovine caudal discs. Spine (Phila Pa 1976). 2006;31(23):2665–73. 17077734 10.1097/01.brs.0000244620.15386.df

[18] Paul CP, Zuiderbaan HA, Zandieh Doulabi B, van der Veen AJ, van de Ven PM, Smit TH, et al Simulated-physiological loading conditions preserve biological and mechanical properties of caprine lumbar intervertebral discs in ex vivo culture. PloS one. 2012;7(3):e33147 10.1371/journal.pone.0033147 22427972 PMC3302815

[19] Haschtmann D, Stoyanov JV, Ettinger L, Nolte LP, Ferguson SJ. Establishment of a novel intervertebral disc/endplate culture model: analysis of an ex vivo in vitro whole-organ rabbit culture system. Spine (Phila Pa 1976). 2006;31(25):2918–25. 17139222 10.1097/01.brs.0000247954.69438.ae

[20] Risbud MV, Izzo MW, Adams CS, Arnold WW, Hillibrand AS, Vresilovic EJ, et al An organ culture system for the study of the nucleus pulposus: description of the system and evaluation of the cells. Spine (Phila Pa 1976). 2003;28(24):2652–8; discussion 8–9. 14673364 10.1097/01.BRS.0000099384.58981.C6

[21] Lee CR, Iatridis JC, Poveda L, Alini M. In vitro organ culture of the bovine intervertebral disc: effects of vertebral endplate and potential for mechanobiology studies. Spine (Phila Pa 1976). 2006;31(5):515–22. 16508544 10.1097/01.brs.0000201302.59050.72 PMC7187957

[22] Korecki CL, MacLean JJ, Iatridis JC. Characterization of an in vitro intervertebral disc organ culture system. Eur Spine J. 2007;16(7):1029–37. 10.1007/s00586-007-0327-9 17629763 PMC2219649

[23] Chan SC, Walser J, Kappeli P, Shamsollahi MJ, Ferguson SJ, Gantenbein-Ritter B. Region specific response of intervertebral disc cells to complex dynamic loading: an organ culture study using a dynamic torsion-compression bioreactor. PloS one. 2013;8(8):e72489 10.1371/journal.pone.0072489 24013824 PMC3755972

[24] Haglund L, Moir J, Beckman L, Mulligan KR, Jim B, Ouellet JA, et al Development of a bioreactor for axially loaded intervertebral disc organ culture. Tissue Eng Part C Methods. 2011;17(10):1011–9. 10.1089/ten.TEC.2011.0025 21663457

[25] Jim B, Steffen T, Moir J, Roughley P, Haglund L. Development of an intact intervertebral disc organ culture system in which degeneration can be induced as a prelude to studying repair potential.20(8):1244–54. 10.1007/s00586-011-1721-x PMC3175835 21336509

[26] Malonzo C, Chan SC, Kabiri A, Eglin D, Grad S, Bonel HM, et al A papain-induced disc degeneration model for the assessment of thermo-reversible hydrogel-cells therapeutic approach. J Tissue Eng Regen Med. 2013; 10.1002/term.1667 23303720

[27] Roughley PJ. Biology of intervertebral disc aging and degeneration: involvement of the extracellular matrix. 2004;29(23):2691. 10.1097/01.brs.0000146101.53784.b1 15564918

[28] Dong, Wang, and, Nam, V., Vo, et al. Bupivacaine decreases cell viability and matrix protein synthesis in an intervertebral disc organ model system. 10.1016/j.spinee.2010.11.017 PMC3056334 21296298

[29] Lim TH, Ramakrishnan PS, Kurriger GL, Martin JA, Mendoza SA. Rat Spinal Motion Segment in Organ Culture: A Cell Viability Study. Spine. 2006;31(12):1291–7; discussion 8. 10.1097/01.brs.0000218455.28463.f0 16721287

[30] Zhu LG, Feng MS, Zhan JW, Zhang P, Yu J. Effect of Static Load on the Nucleus Pulposus of Rabbit Intervertebral Disc Motion Segment in Ex vivo Organ Culture. Chin Med J (Engl). 2016;129(19):2338–46. 10.4103/0366-6999.190666 27647194 PMC5040021

[31] Xulu Yin. The mechanism of BSHXR on nucleus pulposus cells of IDD by regulating Wnt/beta-catenin pathway. Chinese Academy of Traditional Chinese Medicine, 2019.

[32] Zhan JW, Feng MS, Zhu LG, Zhang P, Yu J. Effect of Static Load on the Nucleus Pulposus of Rabbit Intervertebral Disc Motion Segment in an Organ Culture. Biomed Res Int. 2016;2016:2481712 10.1155/2016/2481712 27872846 PMC5107879

[33] Kroeber MW, Unglaub FH, Schmid C, Thomsen M, Nerlich A, Richter W. New in vivo animal model to create intervertebral disc degeneration and to investigate the effects of therapeutic strategies to stimulate disc regeneration. Spine. 2002;27(23):2684 10.1097/00007632-200212010-00007 12461394

[34] Bian Q, Séguin CA, Riley LH, Wang Y, Cao X, Ma L, et al Mechanosignaling activation of TGFβmaintains intervertebral disc homeostasis. Bone Research. 2017;005(001):27–40. 10.1038/boneres.2017.8 PMC5360159 28392965

[35] Xu H, Qiu G, Wu Z, Wang Y, Zhang J, Liu Y, et al Expression of Transforming Growth Factor and Basic Fibroblast Growth Factor and Core Protein of Proteoglycan in Human Vertebral Cartilaginous Endplate of Adolescent Idiopathic Scoliosis. Spine. 2005;30(17):1973–8. 10.1097/01.brs.0000176445.01967.8a 16135988

[36] Weidner N. Intratumor microvessel density as a prognostic factor in cancer. American Journal of Pathology. 1995;147(1):9–19. 7541613 PMC1869874

[37] Yamaguchi T, Goto S, Nishigaki Y, Espinoza Orías AA, Bae WC, Masuda K, et al Microstructural analysis of three-dimensional canal network in the rabbit lumbar vertebral endplate. J Orthop Res. 2015;33(2):270–6. 10.1002/jor.22759 25367593

[38] Xavier LL, Viola GG, Ferraz AC, Cunha CD, Deonizio JMD, Netto CA, et al A simple and fast densitometric method for the analysis of tyrosine hydroxylase immunoreactivity in the substantia nigra pars compacta and in the ventral tegmental area. Brain Research Protocols. 2005;16(1–3):58–64. 10.1016/j.brainresprot.2005.10.002 16310404

[39] Keller TS, Hansson TH, Abram AC, Spengler DM, Panjabi MM. Regional variations in the compressive properties of lumbar vertebral trabeculae. Effects of disc degeneration. Spine. 1989;14(9):1012–9. 10.1097/00007632-198909000-00016 2781407

[40] Adams MA, Dolan P. Spine biomechanics. Journal of biomechanics. 2005;38(10):1972–83. 10.1016/j.jbiomech.2005.03.028 15936025

[41] Sharma A, Lancaster S, Bagade S, Hildebolt C. Early pattern of degenerative changes in individual components of intervertebral discs in stressed and nonstressed segments of lumbar spine: an in vivo magnetic resonance imaging study. Spine (Phila Pa 1976). 2014;39(13):1084–90. 24503691 10.1097/BRS.0000000000000265

[42] Hansson TH, Keller TS, Panjabi MM. A study of the compressive properties of lumbar vertebral trabeculae: effects of tissue characteristics. Spine. 1987;12(1):56–62. 10.1097/00007632-198701000-00011 3576357

[43] Delucca JF, Cortes DH, Jacobs NT, Vresilovic EJ, Duncan RL, Elliott DM. Human cartilage endplate permeability varies with degeneration and intervertebral disc site. Journal of biomechanics. 2016;49(4):550 10.1016/j.jbiomech.2016.01.007 26874969 PMC4779374

[44] Lao Y, Xu T, Jin H, Ruan H, Wang J, Zhou L, et al Accumulated Spinal Axial Biomechanical Loading Induces Degeneration in Intervertebral Disc of Mice Lumbar Spine. Orthopaedic Surgery. 2018;10(1). 10.1111/os.12365 PMC6594506 29436145

[45] Jr RR. Vascular endothelial growth factor (VEGF) and VEGF receptor inhibitors in the treatment of renal cell carcinomas. Pharmacological Research. 2017;120:116–32. 10.1016/j.phrs.2017.03.010 28330784

[46] Xu HG, Ding GZ, Chen XH, Wang H, Wang LT, Chen XW. [Effects of vascular endothelial growth factor vector on vascular buds of vertebral cartilaginous endplate in rabbits]. Zhonghua Yi Xue Za Zhi. 2012;92(7):491 10.3760/cma.j.issn.00376-2491-2012.07.016 22490974

[47] Hong-Guang XU, Chen XH, Ding GZ, Wang H, Wang LT, Chen XW. Effect of pcDNA3.1-vascular endothelial growth factor 165 recombined vector on vascular buds in rabbit vertebral cartilage endplate. Chinese Medical Journal. 2012;125(22):4055 23158142

[48] Rodriguez AG, Rodriguez-Soto AE, Burghardt AJ, Berven S, Majumdar S, Lotz JC. Morphology of the human vertebral endplate. Journal of Orthopaedic Research. 2012;30(2):280–7. 10.1002/jor.21513 21812023 PMC3209496

[49] Bluteau G, Julien M, Magne D, Mallein-Gerin F, Weiss P, Daculsi G, et al VEGF and VEGF receptors are differentially expressed in chondrocytes. Bone. 2007;40(3):568–76. 10.1016/j.bone.2006.09.024 17085091

[50] Pufe T, Mentlein R, Tsokos M, Steven P, Varoga D, Goldring MB, et al VEGF expression in adult permanent thyroid cartilage: implications for lack of cartilage ossification. Bone. 2004;35(2):543–52. 10.1016/j.bone.2004.02.026 15268907

[51] Lantieri LA, Martin-Garcia N, Wechsler J, Mitrofanoff M, Raulo Y, Baruch JP. Vascular endothelial growth factor expression in expanded tissue: a possible mechanism of angiogenesis in tissue expansion. Plastic & Reconstructive Surgery. 1998;101(2):392–8. 9462772 10.1097/00006534-199802000-00020

[52] Chiba K, Andersson GB, Masuda K, Momohara S, Williams JM, Thonar EJ. A new culture system to study the metabolism of the intervertebral disc in vitro. Spine (Phila Pa 1976). 1998;23(17):1821–7; discussion 8. 9762737 10.1097/00007632-199809010-00002

[53] Ariga K, Yonenobu K, Nakase T, Hosono N, Okuda S, Meng W, et al Mechanical stress-induced apoptosis of endplate chondrocytes in organ-cultured mouse intervertebral discs: an ex vivo study. Spine (Phila Pa 1976). 2003;28(14):1528–33. 12865839

[54] Chan SC, Ferguson SJ, Wuertz K, Gantenbein-Ritter B. Biological response of the intervertebral disc to repetitive short-term cyclic torsion. Spine (Phila Pa 1976). 2011;36(24):2021–30. 21343864 10.1097/BRS.0b013e318203aea5

[55] Kim KW, Lim TH, Kim JG, Jeong ST, Masuda K, An HS. The origin of chondrocytes in the nucleus pulposus and histologic findings associated with the transition of a notochordal nucleus pulposus to a fibrocartilaginous nucleus pulposus in intact rabbit intervertebral discs. Spine (Phila Pa 1976). 2003;28(10):982–90. 12768135 10.1097/01.BRS.0000061986.03886.4F

[56] Curatola AM, Moscatelli D, Norris A, Hendricks-Munoz K. Retinal blood vessels develop in response to local VEGF-A signals in the absence of blood flow.81(2):0–158. 10.1016/j.exer.2005.06.001 16011835

[57] Ashina K, Tsubosaka Y, Kobayashi K, Omori K, Murata T. VEGF-induced blood flow increase causes vascular hyper-permeability in?vivo. Biochemical & Biophysical Research Communications.464(2):590–5. 26163262 10.1016/j.bbrc.2015.07.014

[58] Sabatino AD, Ciccocioppo R, Armellini E, Morera R, Ricevuti L, Cazzola P, et al Serum bFGF and VEGF correlate respectively with bowel wall thickness and intramural blood flow in Crohn's disease&nbsp. Inflammatory Bowel Diseases. 2006;10(5):573–7. 10.1097/00054725-200409000-00011 15472517

[59] Kroeber M, Unglaub F, Guehring T, Nerlich A, Hadi T, Lotz J, et al Effects of controlled dynamic disc distraction on degenerated intervertebral discs: an in vivo study on the rabbit lumbar spine model. Spine (Phila Pa 1976). 2005;30(2):181–7. 15644753 10.1097/01.brs.0000150487.17562.b1

[60] Guehring T, Omlor GW, Lorenz H, Engelleiter K, Richter W, Carstens C, et al Disc distraction shows evidence of regenerative potential in degenerated intervertebral discs as evaluated by protein expression, magnetic resonance imaging, and messenger ribonucleic acid expression analysis. Spine. 2006;31(15):1658–65. 10.1097/01.brs.0000224558.81765.56 16816759

[61] Tarkka T, Sipola A, Jämsä T, Soini Y, Hautala T. Adenoviral VEGF-A gene transfer induces angiogenesis and promotes bone formation in healing osseous tissues. Journal of Gene Medicine. 2003;5(7):560–6. 10.1002/jgm.392 12825195

[62] Kaspar D, Neidlinger-Wilke C, Holbein O, Claes L, Ignatius A. Mitogens are increased in the systemic circulation during bone callus healing. Journal of Orthopaedic Research. 2003;21(2):320–5. 10.1016/S0736-0266(02)00134-1 12568965

[63] Kokubo Y, Uchida K, S, Yayama T, Sato R, Nakajima H, Takamura T, et al Herniated and spondylotic intervertebral discs of the human cervical spine: histological and immunohistological findings in 500 en bloc surgical samples. Laboratory investigation. J Neurosurg Spine. 2008;9(3):285–95. 10.3171/SPI/2008/9/9/285 18928227

[64] Yasuma T, Arai K, Yamauchi Y. The histology of lumbar intervertebral disc herniation. The significance of small blood vessels in the extruded tissue. Spine. 1993;18(13):1761–5. 10.1097/00007632-199310000-00008 7694378

[65] Lu XY, Ding XH, Zhong LJ, Xia H, Chen XD, Huang H. Expression and significance of VEGF and p53 in degenerate intervertebral disc tissue. Asian Pacific Journal of Tropical Medicine. 2013;6(1):79 10.1016/S1995-7645(12)60206-5 23317892

[66] Miyamoto T, Muneta T, Tabuchi T. Intradiscal transplantation of synovial mesenchymal stem cells prevents intervertebral disc degeneration through suppression of matrix metalloproteinase-related genes in nucleus pulposus cells in rabbits. Arthritis Research & Therapy.12(6). 10.1186/ar3182 PMC3046513 21054867

[67] Guehring T, Unglaub F, Lorenz H, Omlor G, Wilke H-J, Kroeber MW. Intradiscal pressure measurements in normal discs, compressed discs and compressed discs treated with axial posterior disc distraction: an experimental study on the rabbit lumbar spine model. European Spine Journal.15(5):597–604. 10.1007/s00586-005-0953-z 16133080 PMC3489348

[68] Guehring T, Nerlich A, Kroeber M, Richter W, Omlor GW. Sensitivity of notochordal disc cells to mechanical loading: an experimental animal study.19(1):113–21. 10.1007/s00586-009-1217-0 PMC2899741 19936803

[69] Haschtmann D, Stoyanov JV, Ferguson SJ. Influence of diurnal hyperosmotic loading on the metabolism and matrix gene expression of a whole-organ intervertebral disc model. J Orthop Res. 2006;24(10):1957–66. 10.1002/jor.20243 16917902

[70] Lim TH, Ramakrishnan PS, Kurriger GL, Martin JA, Stevens JW, Kim J, et al Rat spinal motion segment in organ culture: a cell viability study. Spine (Phila Pa 1976). 2006;31(12):1291–7; discussion 8. 16721287 10.1097/01.brs.0000218455.28463.f0

[71] Seol D, Choe H, Ramakrishnan PS, Jang K, Kurriger GL, Zheng H, et al Organ culture stability of the intervertebral disc: rat versus rabbit. J Orthop Res. 2013;31(6):838–46. 10.1002/jor.22285 23456659

