# Supplementary figures and images for: Constant compression decreases vascular bud and VEGFA expression in a rabbit vertebral endplate ex vivo culture model
Source: PLoS One. 2020 Jun 25;15(6):e0234747. doi: 10.1371/journal.pone.0234747 (PMC7316323; doi:10.1371/journal.pone.0234747)

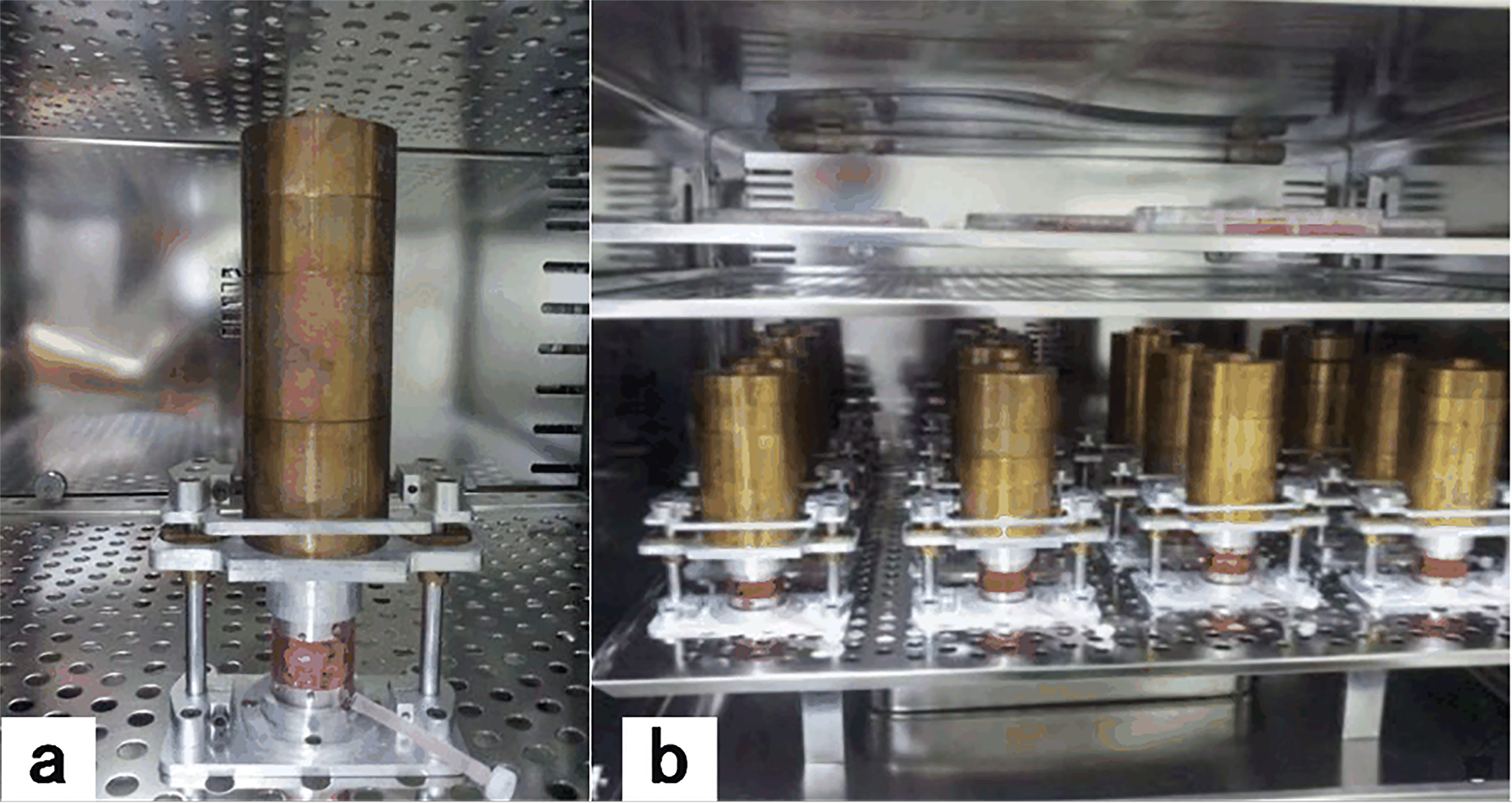

Supplement: S1 Fig — The specimens were maintained in custom-made apparatuses without loading or under a constant compressive load (a); the apparatuses were situated inside a 37°C incubator with 5% CO2 and 100% humidity (b). (TIF) [file pone.0234747.s001.tif]

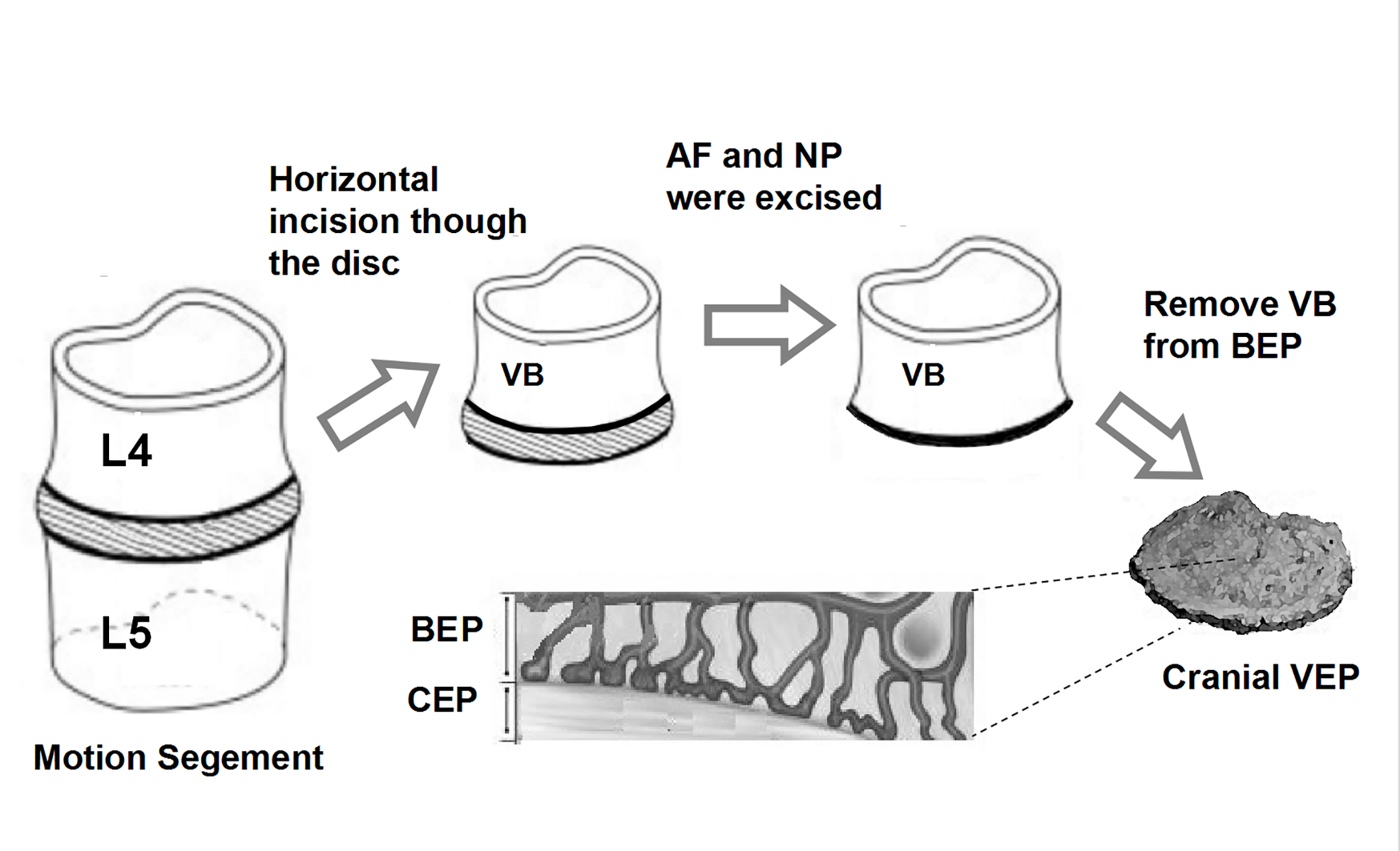

Supplement: S2 Fig — VB: vertebral bone, AF: annulus fibrosus, NP: nucleus pulposus, VEP: vertebral endplate, BEP: bony endplate, CEP: cartilage endplate. (TIF) [file pone.0234747.s002.tif]

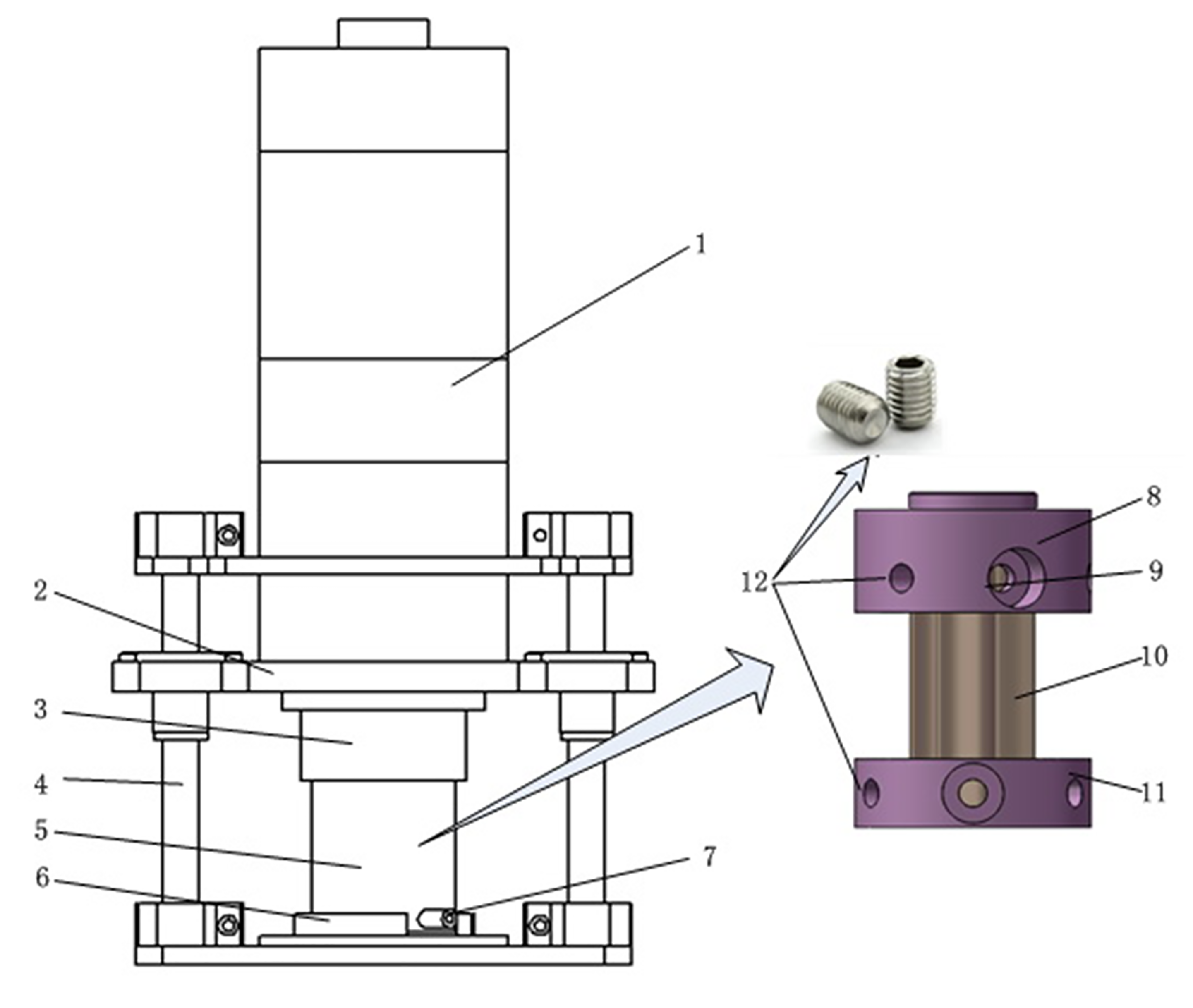

Supplement: S3 Fig — 1. Weight; 2. Loading plate; 3. Gland; 4. Optical axis; 5. Chamber; 6. Chamber fixed base; 7. Outlet; 8. Top pedestal; 9. Fluid level observation hole; 10. IVD motion segments; 11. Base pedestal; 12. Jackscrews. (TIF) [file pone.0234747.s003.tif]
